# “I was referred from the other side”: Gender and HIV testing among older South Africans living with HIV

**DOI:** 10.1371/journal.pone.0196158

**Published:** 2018-04-23

**Authors:** Enid Schatz, Lucia Knight

**Affiliations:** 1 Department of Health Sciences & Department of Women’s & Gender Studies, University of Missouri, Columbia, Missouri, United States of America; 2 School of Public Health, University of Western Cape, Bellville, South Africa; Tulane University School of Public Health and Tropical Medicine, UNITED STATES

## Abstract

South Africa has a Universal Test and Treat (UTT) policy in place to ensure that everyone tests for HIV and can access treatment if they test positive. The aim of this study is to document the pathways that older South Africans who are living with HIV take to access testing and treatment in this context. Despite the aging of the HIV epidemic in South Africa and clear evidence that testing older persons (over age 50) is necessary, very little is known about the circumstances under which older persons test for HIV or their motivations for doing so. In this study, we analyze 21 qualitative, in-depth interviews with women and men aged 50 and over who are living with HIV from two townships outside of Cape Town. Using grounded theory to specify emerging themes, we find similarities and differences between older men and women in their pathways to testing. Men primarily test for HIV when their spouse is diagnosed or in connection with TB testing and treatment. Older women, who are more likely to be widowed or divorced, often test for HIV only when they are symptomatic or not responding appropriately to care for non-communicable diseases. Most importantly, we find that older South Africans do not seek testing as a response to risk. Instead, older men and women test only once they are symptomatic and referred by a provider, or as a result of a partner’s status. Our respondents, particularly the women, expressed “shock” and confusion at learning they were HIV-positive because they do not see themselves as at risk of acquiring HIV. Because the benefits of UTT are greatest with early detection and treatment, older persons’ tendency to test at such a late stage of illness decreases the individual and population level advantages of UTT. More research is needed to understand older persons’ risk and testing behavior so that policy and programs include HIV testing messages that reach this population.

## Introduction

Most HIV messaging in South Africa is targeted toward young persons (15–35 years of age). However, HIV prevalence among those aged 50-plus is 7.1%, which is not significantly different statistically from those 15–24 years old [1]. Although HIV prevalence is highest between the ages of 15 and 49 (18.8%), there are a number of reasons why we should pay attention to HIV risk at older ages. With the rollout of antiretroviral treatment (ART), many individuals are now aging with HIV [2]. In addition, older persons remain sexually active as they age and may engage in sexual behavior that leads to HIV [3], such as extramarital sex, repartnering after divorce and widowhood, limited condom use, and sex with younger persons who are more likely to have HIV [4,5].

The number of older adults who are HIV-infected or at risk of becoming infected is clearly increasing, but older adults remain less likely to test for HIV than younger adults [1,6,7]. In a South African national survey, only 54% of persons aged 50-plus reported having ever tested for HIV, compared to 78% of those aged 25 to 49 [1]. When older persons do test, it is often initiated by the provider because the patient is symptomatic; this occurs at a late stage of HIV infection when ART is less effective [7–9].

At younger ages, testing behavior is fundamentally gendered, with men being much less likely to test than women [10,11]. The gender differences are partly due to how testing has been built into sexual and reproductive health care. Women are offered testing more regularly as part of routine care [12,13]. Among older adults, the gender pattern reverses. Older men are less likely to self-initiate testing than their younger counterparts [14], but older women are much less likely to test than younger women and less likely to test than older men [1,6]. Older women, perhaps even more so than older men, may not see themselves as at risk for HIV [15]. As a result, older women and their providers are less likely to see HIV testing as a key part of routine care, even when they are seeing providers for non-communicable diseases (NCDs) and other conditions [16,17]. Although men’s testing prevalence is also lower at older ages, it is women’s dramatic decrease in testing prevalence that leads us to believe they are falling through the cracks [6].

The ambitious Joint United Nations Programme on HIV/AIDS (*UNAIDS*) target of 90% diagnosed, 90% on ART, and 90% virally suppressed [18] will not be reachable if older persons continue to be ‘‘left behind” [19], i.e., not tested and missing the entry point into the cascade of HIV care. Universal Test and Treat (UTT) is the current policy in South Africa; the aim of this policy is that all individuals know their HIV status and that all HIV-infected individuals are offered immediate access to ART [20]. When HIV-infected individuals adhere to ART, their viral load is suppressed and they are less likely to transmit HIV to their sexual partners [21]. Older persons, however, are not testing at the early stages of HIV, which compromises their health and increases the likelihood of transmission [20–22]. Current data on older persons’ testing behavior is largely limited to questions about lifetime ever-testing. To better understand older persons’ sexual risk behavior and motivations and barriers to testing, the field needs innovative data collection approaches that go beyond standard surveys.

In this paper, we use qualitative data from in-depth interviews with older South Africans who are living with HIV and key informant interviews with health care providers to outline the key pathways to testing among older men and women. We find that both men and women report testing when they are already sick, often in conjunction with being tested for tuberculosis (TB). However, among older men and women, their life course stage at the time of testing differs. We found that men are often brought in for testing because their partners had recently tested positive for HIV, whereas women were mainly unpartnered owing to widowhood or divorce [15]. What was unexpected was that older women seemed no more likely than their male peers to test regularly or as part of routine care, despite having been exposed to routine testing during their reproductive years (antenatal care). Instead, like men, women tested primarily only when advised by a provider because they were symptomatic, and where seeking care for TB or chronic conditions.

## Materials and methods

We explore the experiences related to HIV testing and care among people aged 50 and over living with HIV in Langa and Khayelitsha, two urban, majority Black communities on the outskirts of Cape Town. Although the Western Cape has the lowest overall provincial HIV prevalence in South Africa at 5% [1), the HIV prevalence in Langa and Khayelitsha is closer to 26% [23,24]. The prevalence of HIV is disproportionately high in informal poor urban areas and among Black populations [1]. In Langa and Khayelitsha, we conducted 21 in-depth, semi-structured interviews (IDIs) with older persons living with HIV and nine key informant interviews (KIIs) with health and social service providers in these communities. All data were collected in 2016/17.

This study is part of a larger project (“Addressing the social and health needs of older South Africans living with HIV”) that has received ethics clearance from the University of the Western Cape Research Ethics Committee (UWC REC) (Registration No: 15/7/103) and approval from the University of Missouri via a reliance agreement. Prior to data collection, we also obtained ethical clearance from the Provincial Department of Health of the Western Cape Province and the permission of facility management. All study participants received information about the study and provided informed consent.

The interviews were conducted by a trained and experienced qualitative interviewer who is an older person from a similar community nearby. The IDIs focused on the older persons’ experiences with HIV testing and care and on their experiences with health care utilization more generally. The KIIs focused on the providers’ perspectives on older persons’ access to HIV care and other health services, as well as unmet health and social service needs in the community.

To select respondents for the IDIs, we used a number of non-probability sampling methods, making connections through a survey list from a local NCD research project, as well as convenience sampling and referrals by health workers at HIV clinics. The variation in sampling techniques allowed us to identify respondents who fit the selection criteria of being aged 50-plus and living with HIV. We aimed to sample a similar number of male and female IDI respondents representing a range of ages between 50 and 80 years old. To prevent possible inadvertent disclosure of HIV status, we approached respondents to talk about their health as older people, access to care and support from the community, with no specific reference to their HIV status. Respondents were given opportunity to self-disclose during the interviews (all but one respondent did so) but were never identified as being chosen because of their HIV status to ensure that they did not feel victimized by their inclusion in the study.

The key informant interviews to understand community health and social services available to older persons were conducted with four staff working at the primary health facilities where the respondents in the study sought HIV and NCD care. Additional KIIs, not used in this paper, included one home-based caregiver, two social workers, one senior (old age) club employee, and one employee at the South African Social Security Agency.

The interviewers digitally recorded all interviews (IDIs and KIIs) and then translated and transcribed them into English from isiXhosa. Both authors (Schatz and Knight) analyzed the data, first by reading the transcripts for familiarization and then coding the data for emergent themes. The authors used an inductive grounded theory approach [23,24] to identify the themes relevant to HIV testing. The authors discussed themes and related data with the interviewer (NC) as a means of member checking, given her relationship with the community. Next, the authors used a deductive analytic approach to code the IDIs and KIIs. Finally, the IDI themes were compared by gender to understand how the pathways to HIV testing are similar and different between the men and women interviewed. Each IDI was identified by the location (L = Langa, K = Khayelitsha), OP for Older Persons, and study ID number. Each KII was identified with KII, the location (L = Langa, K = Khayelitsha), and interview number.

## Findings

Importantly, we found that the primary pathway to HIV testing among our respondents was a provider-initiated test because of their own or their partners’ health status. All but two of the men in our study had tests that were provider-initiated either because (a) their spouse was sick or tested and partner testing was suggested, or (b) because they themselves presented as sick and thus were tested–often for both TB and HIV simultaneously. Several of the men diagnosed in the last five years were in their 50s and reported that they had partners who were also living with HIV. Because female partners are likely several years younger than the men [25], the wives were likely tested and diagnosed with HIV in their 40s or 50s. Only two respondents reported wives being tested in connection with pregnancy. Many of our female respondents had experienced widowhood or divorce, but that did not necessarily motivate them to get HIV tested. Like men, women were likely to test only when sick and prompted by a provider. And, although fewer women than men reported having been sick with TB when they tested for HIV, the majority of women did report having other co-morbidities (e.g., high blood pressure, diabetes) and being symptomatic at the time of their HIV test. Women in particular reported that prior to HIV testing, they assumed they were not at risk for HIV and were thus shocked when diagnosed. In the next section, we present quotes from IIDs and KIIs that demonstrate each of these issues.

### Timing of testing

Respondents all chose to focus on the HIV test that informed them they were positive; the interview protocol did not include probes for previous testing experiences. Although not all of our respondents gave the year in which they tested positive, nearly half of our respondents reported it was within the last five years (2012–2016). One of these respondents had been diagnosed within the last six months. Eight respondents identified a date 10 to 12 years ago when they learned their HIV status was positive or said their positive test had been “many years ago.” Among respondents that had tested positive in the last five years, the women were in their late 50s to late 60s and the men were in their 50s. Thus, although some of our respondents learned they were living with HIV in their 40s and survived into older ages on ART, the majority of our respondents did not learn their HIV status until their 50s. One key informant confirmed that “we have quite a few clients that [are] testing positive now over the age of sixty, as well” (KII-L4, Health worker). The fact that most testing reported by our respodents took place in rheir 50s suggests that this testing was not a part of routine sexual and reproductive health services, such as antenatal care.

### “I only came to know about HIV when my wife got sick”

Among couples, the wives of our respondents most often tested first and prompted men’s testing. But not always. In a limited number of cases men were tested first and their female partners followed. A 54-year-old man whose wife was also HIV-positive and on ART said,

“My wife had gone there and she said I’m wanted at the clinic; she is also on ARV treatment; they wanted me there and they found that I’m also HIV-positive; so, both of us drink the same pills but she was the first to use them”

(LOP-07).

A 67-year-old male respondent explains how he was invited to test because of his wife’s diagnosis. He said a nurse requested that he test in part because he was sick, but also “another thing is that my wife at that time was pregnant, she also discovered during her pregnancy at the time of delivery that she’s HIV-positive, so it became necessary for her to be treated” (KOP-04).

Another 64-year-old male respondent explained the circumstances of his test:

“I had to attend treatment for High Blood [pressure], I only came to know about HIV when my wife got sick and then my wife fell ill and we brought her here to the clinic and she was tested and she was diagnosed with HIV and as someone living with her I had to be tested as well and as she was diagnosed HIV-positive then I was found to be affected [infected] too and I also was treated in that way because it was discovered in her and we were then put on treatment”

(KOP-07).

One male respondent, aged 57, highlighted a less explicit request from his wife for him to test:

“I’ve got a wife that I come with [to test] who would say, ‘Let’s go to the clinic’, [I’d ask] ‘What for?’ because I’m not used to coming to the clinic and I had not been diagnosed with TB yet at that time…[I’d ask] ‘What for at the clinic?’ and she would say ‘No, I have been told to bring you with [me] to the clinic’ and I would say ‘What for at the clinic,’ then one day I saw some people arriving, these counsellors who said ‘We would like you to come to the clinic for a blood test’ and I could see that there is something that she has been told”

(KOP-01).

Two men explicitly stated that they had tested positive before their wives. One man in his 50s who was tested because he was positive for TB said,

“it was found out that I have HIV and then they asked for my wife and she wasn’t with me and then she later had to come with the children as well for testing, … they were both tested and were also diagnosed HIV-positive”

(KOP-05).

In another couple, the husband’s HIV testing, and wife subsequent testing, revealed that he and his wife were sero-discordant. This 50 year-old man narrated what happened after he tested positive:

“When I got home I told my wife and she said it’s a good thing that I went and I said she must also go. She came here and came home with her results saying there’s nothing wrong and I said but you must go every three months and she agreed that she was also told [this at the clinic]”

(KOP-08).

He and his wife now attend the clinic every three months, him to pick up his ART and her to test.

### “At the time I was not well and being checked for TB…”

TB and HIV are closely linked, and their care is often integrated. As such, it is common practice to test for HIV if someone presents with TB symptoms [26,27]. For this reason, it is not surprising that in several cases our male respondents reported that they were tested for both HIV and TB when they came to a clinic with coughing or other TB-related symptoms. As one 60-year-old respondent said,

“At a time I was not well and being checked for TB here then I was simultaneously checked for HIV and I was told that I have it… I came here because of ill-health and I went to a doctor in Mfuleni and he gave me medication and on the same day a young man arrived, then he said I must go to the clinic, at the clinic everything would be tested”

(KOP-02).

Another respondent in his mid-50s said,

“I first contracted TB and I wasn’t aware of it… when I went upcountry at home I observed that I’m losing weight and I would think why am I losing weight, then I went to another clinic there and they didn’t tell me what I’m suffering from and my weight was going down and then I decided to come back to Cape Town quickly and when I arrived I came to the clinic and they injected me and various things they did and they then told me that I have HIV plus TB”

(KOP-03).

However, not all respondents reportd HIV testing at the beginning of their TB treatment. Asked if he came on his own for HIV testing or if he was referred, one man in his 50s said,

“No it started from TB, when I was on TB treatment, so when I asked about the TB treatment then it was said I’m near the end, then they asked me if I ever went for a [HIV] test and I said I haven’t been and I asked where the test is done and one of them took me inside and I had a test there and it was found out that I have HIV”

(KOP-05).

Data from the KIIs confirm older persons usually get tested only when they are provider-referred or symptomatic, rather than voluntarily or part of routine testing, and that TB is a key entry point to HIV care. As one health worker said,

“There is not many old people coming specifically to ask for HIV [tests], they come maybe because they’ve been coughing for more than a month, even then when they come they are not coming for HIV, they are coming to test for TB and now the protocol for somebody that has been coughing for more than two weeks, then if they come then we screen them for TB and then we ask them to get tested for HIV because it goes together but we may not force them, we just offer them the test”

(KII-L2, Health worker).

Reflecting on why testing often occurs later among older adults, one health worker said, “Some of them refuse totally [to test for HIV] up until they are discharged [from TB care] but when they come back they are too sick, then they have no choice.” When asked if it is men or women who more commonly act this way, the health worker responded, “Most[ly] it’s men, some of the females are also refusing, but mostly it’s men” (KII-K1, Health worker).

### “I didn’t request anything [testing], I was very weak, I had diarrhea”

Among our ten female respondents, only three reported they sought out testing. One woman reported testing because “I just thought that I should go and have a test” (LOP-04). A second woman reported that she was tested because she was gang-raped. The third woman was widowed in the 1980s and did not think she was at risk, but eventually she sought testing because she was feeling sick, losing weight, had TB, and was encouraged by a friend to get HIV tested. The remaining seven female respondents were tested when a provider suggested they do so. None of the ten women were diagnosed in the context of routine antenatal care, and all were tested later in life when they had other health conditions. Half of the women had TB, but the majority also had at least one non-communicable disease (heart disease, diabetes) or other chronic condition like high blood pressure, arthritis, or asthma. It is important to highlight that nearly all of the women were tested for HIV in connection with on-going care for another chronic condition.

For example, a 61-year-old widow was in care for high blood pressure when she tested in 2013. As she explained,

“Yes it was diagnosed on the other side at OPD at the time that my husband died; HIV was diagnosed when I was already receiving treatment for High Blood [Pressure]…At the time that I was very sick, my eye was quite red and swollen [*At the time you were diagnosed with High Blood*?] No at the time HIV was diagnosed and it was because of that, that I came here because it was red and swollen and here I was given a letter and I was admitted to Tygerberg Hospital”

(KOP-09).

Another widow (62 years old) got tested when she sought care because, as she said, “my body was very painful and there’s no part of my body that was not getting this pain.” She was diagnosed with TB via an X-ray and did not ask for an HIV test at the time. She went on to say, “I wasn’t looking out on any other thing… they [the health workers] decided [to test for HIV] … I never said anything about it, they saw it…I had gone there for TB” (LOP-02).

A 60-year-old unmarried woman was already enrolled in care for high blood pressure, arthritis, and TB in 2015 when she said, “I was tested here because I was being treated for TB and then the doctor asked if I want a test and I agreed and thereafter he told me that I’m HIV-positive” (KOP-13). A 64-year-old respondent explained how she came to test after she separated from her husband:

“My health problem started with my experiencing dizziness after I had parted with my husband. I became dizzy and gradually became weak and started having diarrhea and I decided to go to the clinic … I was anxious, weak, and unable to walk and then I was diagnosed as being HIV+ here in Cape Town … I was being treated for HIV and TB, they were together”

(KOP-12).

An older widow, aged 71, was diagnosed just two months before being interviewed and said she had never been tested previously (KOP-11). She had gone into the clinic for a chest problem when the doctor suggested an HIV test; she agreed to the test and was surprised to learn she was HIV-positive.

### “I wasn’t someone who is promiscuous; I was focusing on one person…”

While most of the men were married and living with HIV-positive partners, none of our female respondents reported living with a spouse or partner, either currently or when they were tested. Four reported being widowed, four were divorced or separated, and two did not mention a partnership. As older women, many of whom had not been regularly sexually active for some time, they did not see themselves as being at risk and thus did not seek out testing on their own. One of the few older women who chose to test was the 58-year-old, unmarried woman who was gang raped.

When one 62-year-old widow was asked if she remembered when she was diagnosed, she said, “I can’t remember because I just thought it was the things of Diabetes and I didn’t move fast to have myself tested” (LOP-02). A 59-year-old woman who was widowed for thirty-plus years, but tested positive in 2004, seemed confused about how she came to be positive. She said, “Being this old where could I have got it from … I wasn’t someone who is promiscuous; I was focusing on one person. I was heartbroken but I’m right now” (LOP-03). She went on to say that when she was counseled, they told her about the ABCs (i.e., abstinence, be faithful, use condoms). But, her response was “and I said to them that ‘sex is not something that I care for, [and you tell me] if I’m going to have sex with someone I must use a condom.’ I don’t even have a partner anymore, I’m just sitting alone.”

The health workers also confirmed that older people, and maybe particularly older women, do not see themselves as at risk and thus only get tested when referred or seeking care for something acute. As one health worker said, “They didn’t ask to be tested, the older one yes because they wouldn’t expect … I wouldn’t want to find out at sixty that I’m positive which means they’ve never checked regularly over the time.” When asked how older persons in particular react to an HIV diagnosis, she said their normal reaction is that of being “Shocked, everybody is shocked, disbelief” (KII-L4, Health worker).

## Discussion

In general, men have been less likely to test for HIV than women [1,13]. And, among older men and younger men, we see a similar pattern: both seem likely to seek services only when they are symptomatic and may not properly practice prevention [8,10,14]. In our anlaysis, two pathways to HIV testing emerge for men. First, the data above reveals how wives, who are likely in their 30s, 40s or 50s, are often linked to care and HIV-tested as part of routine antenatal testing, which then results in their male partners getting tested [10,11,14]. Tuberculosis testing and care is the other key pathway through which men are linked to HIV testing [26]. This pathway reflects intentional policy and programs [28,29]. Older men (and likely younger men, also) who present with TB symptoms are routinely tested for both TB and HIV [26]. However, if men present with other illnesses, they are not necessarily tested for HIV until highly symptomatic (8). In sum, men are most often linked to testing through their provider or partner. But, as the data above reveal, wives may also use surreptitious means to get men to the clinic to test, which reminds us that even in the era of ART, disclosure remains complex.

The novel and unanticipated finding in our study is that once women age out of routine testing, their likelihood to self-initiate testing is not any greater than it is for men. This is contrary to expectation because women have typically had more engagement with health care services, including routine testing in their reproductive years as part of sexual and reproductive health care services [12,30–32]. This greater healthcare engagement does not appear to translate to older women regularly testing for HIV [6]. This may be in part due to women’s life-stage, which differs from men. The majority of our female respondents were not partnered when they tested positive for HIV; and as a result, they perceived their HIV risk to be low. Not being partnered lowers risk perception and thus reduces the probability of seeking or being offered HIV testing. However, this view of risk as determined by promiscuity, multiple/regular partnerships, or partner’s lack of fidelity, may be outdated.

Another key pathway to HIV testing for women is provider-initiated testing when they are symptomatic and at advanced stages of HIV infection. In fact, this may be the main pathway for older women, but older women are particularly invisible and thus likely to fall through the cracks. Having TB or another advanced, non-responsive chronic condition is what eventually led providers to link older women to HIV testing and care. But providers, too, seem reluctant to test older women for HIV unless symptoms are advanced. Providers often do not see older women as at risk, nor do they readily acknowledge that older persons may become newly infected. Thus, providers may overlook or misinterpret early signs and symptoms, not suggesting testing until the client is very ill [17,33]. Older women are more likely to be engaged with the health system than older men because health seeking patterns are gendered [8,13,34] and because women have high rates of NCDs [35]. Despite their engagment, the view that older women are not “at risk” appears to negatively bias both providers’ and clients’ likelihood of suggesting testing.

### Reframing HIV risk and testing for older Africans

Our findings suggest three key issues: (1) the need to incorporate life course theory and analysis into work on aging and HIV, with particular attention to the different life stages that older men and women are likely to be in at ages 50 and over (e.g., see important work by Mojola and colleagues [15]); (2) the need for older men and women and their providers to rethink the categories and trajectories of risk, which should prompt earlier testing than is currently happening; and (3) the need to link older adults to HIV services in a more routine fashion.

Gendered life course theory, which highlights linked-lives and shifting risk and relationships [36], may be a stronger way of conceptualizing and understanding older persons’ experiences of HIV risk and testing. As outlined in Mojola et al., HIV vulnerability itself varies as people age because of changes in risk and protective strategies and changes in the environments in which people live [15]. Importantly, vulnerability is shaped by gender, with older men and women experiencing life course stages at different ages and with different positions in their families and communities [37,38]. Shifts for men and women include changes in marital status, access to sexual and reproductive health care, menopause, decreasing sexual desire, returning from migrant labor, pension receipt, and co-morbidities related to aging (e.g., NCDs).

In our study, the men were younger (on average) and reported remaining sexually active longer than the women. We also know men may be more likely to seek out extra-marital partners at older ages [4]. This has the potential to introduce risk into the relationship, which mirrors the epidemic in younger populations [39,40]. If men have younger female partners or secondary partners, their relationships could also increase the risk of secondary transmission to older women [39]. This is especially troubling in light of our finding that women perceive themselves to be at lower levels of risk. Older women may also be more likely to trust their partners and less likely to take precautions [1]. Indeed, other data show that condom use is lower and less consistent within relationships in older populations [1,41]. Our data highlight the continued importance of tracking the ways that relationships and trust may reduce risk perception and therefore the drive to test for HIV.

Another relevant life-course and linked-lives issue is the important role that older persons, particularly older women, play as caregivers for grandchildren [38]. As visible caregivers to children and respected elders, older persons’ willingness to test is an important model for younger generations [42,43] to emulate. Ignoring HIV in in older adults, especially in light of their caregiving role, could have important negative impacts. Because a positive HIV status requires resources and may exacerbate poverty [44], the effect that older adults testing behavior has on younger people has implications not only for health but also the transmission of poverty [45].

Next, we argue that as the HIV epidemic ages, the standard categories and trajectories of risk highlighted in HIV research and policy need to be rethought [13,46]. Older persons, and particularly older women, could be sexually inactive but infected many years prior. As such, their current sexual activity may be a poor proxy of risk. Further, the likelihood of older persons’ HIV symptoms being missed or interpreted as other diseases is high [33], especially for older women who are not seen by themselves or providers as being at risk [17]. And because older adults have such a high likelihood of comorbid conditions (e.g., high blood pressure, diabetes, cancer), the likelihood of symptoms being misinterpreted or missed may be higher [33]. For these reasons, it is important to explore the types of HIV-related risk that older persons experience, both in terms of incidence and timing, as well as how those coincide with or are masked by symptoms of aging or other non-communicable diseases. Certain co-morbidities may increase one’s immulogical risk of acquiring HIV, and may help to mask symptoms and decrease the likelihood of being tested for HIV [33]. The South African public health system aims to integrate care for NCDs and HIV, but it is not yet a reality. Achieving this aim may be a way to increase the likelihood that older persons are tested for HIV more routinely and can access care for both conditions in a more streamlined fashion [47]. Integrating NCD and HIV care will require retraining health care professionals to approach patients’ care more holistically, including to suggest HIV testing on a more routine basis, particularly if there are any early symptoms or the older person has not been tested in the previous five years.

At the same time, beyond policy, there needs to be a recognition that aging does not exempt one from the more traditional forms of risk. At younger ages, the majority of women get HIV tested as part of antenatal and reproductive health services [12,32]. As women age out of their reproductive years, they may have more limited interactions with health services and fewer provider conversations about sexual health. However, HIV risk remains. Although only one female respondent said that her reason for testing was that she had been raped, rape is a major public health issue in South Africa [48]. Athough much of the research focuses on younger women [49], the reality is that older women also fear for their safety in various contexts. A number of women in our study mentioned fear of rape as affecting their decisions to go to certain areas and their willingness to let people into their homes. Their fear, as well as the reality of prevalent gender-based violence in South Africa, speaks to another aspect of HIV risk that is likely to be overlooked as women age [48,50]. For these reasons, we argue that providers need to talk to older persons about sexual health and offer HIV testing more routinely, not just when women are symptomatic.

### Limits of current data

Our qualitative data, like most survey data, focused on the results of our respondents’ most recent HIV test. Given that we knew the HIV status of our respondents, we did not need to ask about ever-testing. Along with most recent test, ever-testing is the other key question that surveys ask. While we were able to gain some information about the motivations for and circumstances around respondents’ most recent (and meaningful) test, we did not ask about testing history, such as whether the most recent test was their first test or whether prior antenatal care (for female respondents) had included testing. About half of our respondents were able to give the year of their HIV-positive test, as it was clearly a meaningful moment in their lives. On the other hand, a number said things like, “it was many years ago” or “long ago,” which points to the complexity of remembering, even in detailed conversation, when an event took place. To address these issues, we believe a new, life history calendar tool is needed that will collect testing history and associated motivations in a format where respondents can more precisely remember occurrence, timing, and sequencing of life events [51,52]. Importantly, a life history calendar tool with domains on sexual relationships and engagement with the health system could address a number of the issues outlined in our study. A life history calendar that focuses on older persons’ health and risk could explore trajectories that lead to testing and could also follow up on why older persons do not see themselves at risk, even when they report behavior that in younger populations might be regarded as risky.

Our study has a number of limitations that may affect the generalizability of our findings, even among older persons in urban communities in Cape Town. First of all, all of the individuals with whom we spoke knew they were living with HIV and were on ART. Thus, they clearly had been tested for HIV and had linked to care. Our sample was a convenience sample, and a number of the respondents were found through HIV clinics. Thus, not only were they individuals who had been linked to care after being tested for HIV, but they were individuals who continued to see the value of HIV care. This may make their testing experiences different from other older persons who were either tested too late for ART or were so demoralized by their testing results that they did not remain linked to care. Additionally, more qualitative work on risk, norms, and perceptions is needed [6,43]. A key addition would be focus group interviews to assess the normative perceptions of HIV testing among older persons across a variety of high prevalence settings (e.g., urban vs. rural). Further, our small scale study only focused on individuals currently living in Khayelitsha. Yet, there are important urban/rural differences in residence and access to care that are related to historically structured migration patterns. Thus, it is essential that future research explores the ways that geographic locations (and movements between them) affect older persons’ access to health care, and HIV testing and care in particular.

Our sample of men were on average younger than the sample of women, which might have affected the finding that women were, on average, testing at older ages than men. We also had limited information about older men’s reaction to having an HIV-positive partner and the negotiation that occurred around accessing the clinic and HIV testing. Although we suggest men may connect to testing through their younger wives, we also realize there is an equally important need to rethink partner disclosure or stigma that affects men’s access to services. Not doing so may result in further delays or problems with men accessing HIV care [13]. While disclosure did not appear to be problematic in our study area, perhaps in part owing to reduced stigma and increased ART availability, partner disclosure remains a critical step in linkage to care [53].

Overall, the work suggests the need to better understand how, when, and why older persons do and do not test for HIV. Focusing on trajectories and transitions in older ages will provide insight into key factors as well as possible intervention points. As the HIV epidemic ages and UTT becomes a key element of not just treatment but also prevention, ensuring that older persons are not “left behind” or missed by testing programs and policies will be crucial to meeting UNAIDS’ 90-90-90 target.
